# Comparison Between Dichloroacetate and Phenylbutyrate Treatment for Pyruvate Dehydrogenase Deficiency

**DOI:** 10.3389/bjbs.2022.10382

**Published:** 2022-05-19

**Authors:** Patricia Karissa, Timothy Simpson, Simon P. Dawson, Teck Yew Low, Sook Hui Tay, Fatimah Diana Amin Nordin, Shamsul Mohd Zain, Pey Yee Lee, Yuh-Fen Pung

**Affiliations:** ^1^ Division of Biomedical Science, Faculty of Science and Engineering, University of Nottingham Malaysia, Semenyih, Malaysia; ^2^ Faculty of Medicine and Health Sciences, University of Nottingham, Nottingham, United Kingdom; ^3^ UKM Medical Molecular Biology Institute (UMBI), Universiti Kebangsaan Malaysia, Kuala Lumpur, Malaysia; ^4^ Institute for Medical Research (IMR), National Institutes of Health, Shah Alam, Malaysia; ^5^ Department of Pharmacology, Faculty of Medicine, University Malaya, Kuala Lumpur, Malaysia

**Keywords:** E1a, lactic acidosis, inborn error of metabolism, mitochondrial disease, PDHA1

## Abstract

Pyruvate dehydrogenase (PDH) deficiency is caused by a number of pathogenic variants and the most common are found in the *PDHA1* gene. The *PDHA1* gene encodes one of the subunits of the PDH enzyme found in a carbohydrate metabolism pathway involved in energy production. Pathogenic variants of *PDHA1* gene usually impact the α-subunit of PDH causing energy reduction. It potentially leads to increased mortality in sufferers. Potential treatments for this disease include dichloroacetate and phenylbutyrate, previously used for other diseases such as cancer and maple syrup urine disease. However, not much is known about their efficacy in treating PDH deficiency. Effective treatment for PDH deficiency is crucial as carbohydrate is needed in a healthy diet and rice is the staple food for a large portion of the Asian population. This review analysed the efficacy of dichloroacetate and phenylbutyrate as potential treatments for PDH deficiency caused by *PDHA1* pathogenic variants. Based on the findings of this review, dichloroacetate will have an effect on most PDHA1 pathogenic variant and can act as a temporary treatment to reduce the lactic acidosis, a common symptom of PDH deficiency. Phenylbutyrate can only be used on patients with certain pathogenic variants (p.P221L, p.R234G, p.G249R, p.R349C, p.R349H) on the PDH protein. It is hoped that the review would provide an insight into these treatments and improve the quality of lives for patients with PDH deficiency.

## Introduction

Inborn errors of metabolism can result in various metabolic deficiencies, ultimately impairing growth and development process (1). One such inheritable defect occurs in the *PDHA1*, a X-chromosomal gene encoding pyruvate dehydrogenase (PDH). In the mitochondria, PDH forms a complex (PDH complex) with two other enzymes to help convert carbohydrate to energy. But certain individuals may carry a pathogenic variant of *PDHA1*, altering the function of the PDH enzyme, and giving rise to a rare disease characterised by reduced energy production (2).

Individuals with PDH deficiency are often prescribed a ketogenic diet, which contains high fat but low carbohydrate (3, 4). With this diet regime, patients obtain most of their metabolic fuels *via* fatty acid oxidation, rather than carbohydrate catabolism. As a result, PDH deficiency can be partially alleviated; though never completely resolved (5). Thus, potential treatments such as dichloroacetate and phenylbutyrate may be an alternative option to the ketogenic diet.

Dichloroacetate has been prescribed to treat several types of cancer such as colorectal cancer, liver cancer and possibly head and neck squamous cell carcinoma (6–8). Cancer patients use dichloroacetate because it reduces signal transduction by the hypoxia inducible factors, which in turn reduces PDH complex activity and minimise the energy dependence on glycolysis in cancer cells (6). Phenylbutyrate was used in maple syrup urine disease, where it acts on the kinase of the branched chain α-ketoacid dehydrogenase, a member of the same family of PDH kinase (9, 10). Phenylbutyrate prevents the PDH kinase from phosphorylating the PDH complex, allowing the complex to remain active.

This review provides an overview of the cause, clinical manifestation, diagnosis and the current known treatments for PDH deficiency due to *PDHA1* pathogenic variants. Additionally, a detailed review of the treatment efficacy of dichloroacetate, phenylbutyrate or a mix of both. It is hoped that this review will create awareness on this inherited disease and shed light on potential treatments so as to improve the quality of lives in PDH deficiency patients.

## Carbohydrate Metabolism Pathway

**FIGURE 1:**
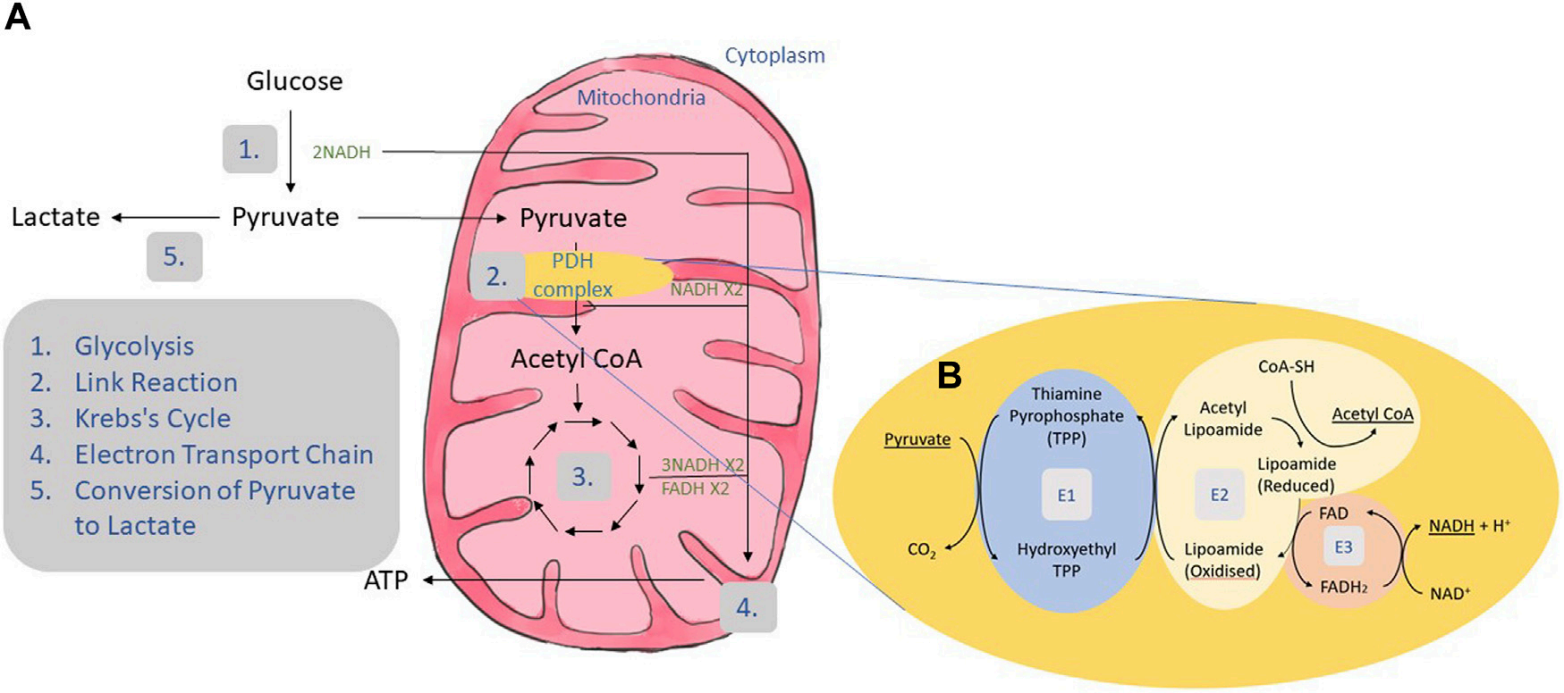
Carbohydrate metabolism. **(A)** Carbohydrate is broken down to glucose. Glucose undergoes the process of glycolysis where it goes through several enzymatic reactions to produce two molecules of pyruvate and two molecules of NADH (1). Glycolysis is followed by link reaction where each pyruvate will be converted to acetyl CoA and NADH (2). The link reaction was mediated by a group of enzymes called the PDH complex. Acetyl CoA produced in the link reaction enters the Krebs cycle generating 3 NADH and 1 FADH (3). All the NADH and FADH produced were transported to the electron transport chain located at the mitochondrial matrix to produce ATP (4). Pathogenic variants in the PDH complex may result in PDH deficiency and reduce the activity of the complex. Due to these pathogenic variants, pyruvate will be converted to acetyl CoA at a slower rate, affecting the subsequent reaction. The excessive pyruvate will build up and be converted to lactate instead (5). **(B)** The process of link reaction starts with the removal of one carbon from pyruvate by PDH (E1) resulting in hydroxyethyl which joins together with thiamine pyrophosphate (TPP) to produce hydroxyethyl TPP and release carbon dioxide. Dihydrolipoyl transacetylase (E2) transfers the hydroxyethyl group to oxidized lipoamide. This causes the acetyl group to be transferred to coenzyme A (CoA) forming acetyl CoA. FAD bound to dihydrolipoyl dehydrogenase (E3) oxidizes the reduced lipoamide by accepting an electron to produce FADH_2_. The electron is donated again to NAD^+^ resulting in the production of NADH and H^+^.

As shown in Figure 1A, carbohydrate metabolism involves four main processes: glycolysis, the link reaction, the Krebs’s cycle and the electron transport chain (2).

In link reaction, pyruvate is converted to acetyl CoA by the PDH complex, which comprises of three enzymes: PDH (E1) which is made up of two α subunits and two β subunits; dihydrolipoyl transacetylase (E2); and dihydrolipoyl dehydrogenase (E3) (Figure 1B).

The PDH complex is activated by PDH phosphatase and inactivated by PDH kinase (2). PDH kinase inactivates the PDH complex by phosphorylating it at one of three serine residues namely Ser264, Ser271 and Ser203 on the α-subunit of the PDH enzyme (11). On the contrary, activation of the PDH complex is achieved by dephosphorylation of the same serine residues by PDH phosphatase. There are four isoforms of PDH kinases reported so far, each with different tissue-specific localization. For example, PDH kinase 1 is located in the heart and skeletal muscle, PDH kinase 2 is ubiquitously expressed in all tissues, PDH kinase 3 in testis, kidney and brain, and PDH kinase 4 is mainly expressed in the skeletal, heart tissue and pancreatic islet (11).

The incidence of PDH deficiency can mostly be attributed to defects in the E1 α-subunit of PDH, which slows down the conversion of pyruvate to acetyl CoA. Consequently, pyruvate is converted *via* another route to lactate, while lower amounts of acetyl CoA are produced. This affects the subsequent Krebs’s cycle and electron transport chain, decreases energy production and finally leads to impaired growth and development.

## Cause, Clinical Manifestation of Pyruate Dehydrogenase Deficiency due to Pathogenic Variations on *PDHA1* Gene

Based on a study by Sofou et al., 15 out of 19 patients suffering from PDH deficiency had prenatal disease onset (12). Defects within the E1 α-subunit of the PDH enzyme arise from nonsense/missense and deletion/insertion pathogenic variants in the *PDHA1* gene found on the X chromosome (13, 14). A study with 130 patients from 123 unrelated families by Lissens et al., found that most female patients have deletion/insertion pathogenic variants occurring in exons 10 and 11; while most male patients manifest nonsense/missense pathogenic variants occurring in exons 3,7,8 and 11 (13). Based on its inheritance pattern, it is assumed that female patients tend to live longer although they may develop cognitive impairment as they grow (15). This was supported by a mutant murine model with an interruption on the *PDHA1* gene. Male embryos died prenatally while the female mice had reduced cell proliferation, migration and differentiation of neurons (16).

Meanwhile, cognitive impairment is reported to be caused by the accumulation of lactate which can lead to congenital lactic acidosis, a common symptom shown for patients carrying the pathogenic variant of *PDHA1* gene (17). Due to excessive lactate conversion, fewer molecules of ATPs are produced, resulting in the malformation of the brain (5). These malformations includes cerebral atrophy, asymmetric ventriculomegaly, dysgenesis or agenesis of the corpus collosum, T2-weighted hyperintensities, periventricular cyst and/or intraventricular septation and hyporotation of the hippocampus (18). These would affect motor and cognitive function. Other manifestations include developmental delay, hypotonia, spasticity, dysplasia, pachygyria and Leigh’s syndrome (2).

## Diagnosis of Pyruvate Dehydrogenase Deficiency due to *PDHA1* Pathogenic Variant

PDH deficiency is diagnosable during the prenatal period using invasive and non-invasive manners. The invasive method begins with sampling from the amniotic fluid, chorionic villi, amniocytes or chorionic villi cells. DNA from these samples is then extracted, amplified and sequenced. The DNA sequences of a subject is subsequently compared with online databases to search for substitution, deletion and pathogenic variants (19). The same method can also be used to detect for PDH deficiency postnatally. In this case, samples were taken from the blood, fibroblasts or other tissues (19). In contrast, non-invasive methods use magnetic resonance, this includes magnetic resonance imaging and magnetic resonance spectroscopy. Magnetic resonance imaging is used to spot for any brain malformation (20). Foetuses may show increased cerebrospinal fluid and decreased cortical sulcation and gyration (20). This is usually followed by magnetic resonance spectroscopy that can be used to observe changes in lactate and pyruvate (20, 21). Patients showing a doublet at 1.33 ppm for lactate and a single peak at 2.37 ppm for pyruvate may imply PDH deficiency.

As an initial symptom of PDH deficiency, lactic acidosis is detected with examining the patient’s lactate concentration by taking blood (venous or arterious) or the cerebrospinal fluid before and after a carbohydrate-rich meal. Venous blood containing basal lactate levels over 2.5 mmol/L and atrial blood samples over 2 mmol/L may indicate PDH deficiency (2). Other sources suggest that blood lactate concentrations in the range of 3–17 mmol/L and pyruvate concentrations ranging from 0.27 to 0.81 mmol/L would indicate PDH deficiency (22). Whereas, cerebrospinal fluid containing concentrations over 2.5 mmol/L may also indicate PDH deficiency. After obtaining the lactate and pyruvate concentrations, these values were usually ratioed for further confirmation. A lactate to pyruvate ratio of less than 5 mmol/L may indicate PDH deficiency (2). Capillary, arterial or venous blood gas can also be used to detect lactic acidosis. A study by Gupta and Rutledge shows that the serum lactate in capillary gas may rise up to 8.9 mmol/L whereas the venous gas may rise up to 9.3 mmol/L (23). Arterial blood gas and serum electrolytes can also be used to calculate anion gap. A normal range anion gap lies in between 4 and 12 mE/L. Values over 12 mE/L indicate metabolic acidosis (18). These methods may only suggest for PDH deficiency as there could be other diseases that elevates the lactate concentration within the blood.

Another diagnostic test for PDH deficiency compares the proline- or alanine- to leucine ratio in dried blood spots (22). The concentrations for the proline, alanine and leucine may be measured using a mass spectrometer (22). Elevations of alanine and proline will indicate an increase of pyruvate and lactic acid. Alanine levels will be impacted by an increase in the transamination of the excess pyruvate whereas proline will increase due to the inhibition of proline oxidase by lactic acidosis (22). Inhibition of proline oxidase will prevent proline from degradation and hence explaining its elevation in sufferers. For reference, leucine is used as a control as leucine is impacted by the glycolytic pathway.

Immunodetection such as western blot and immunocytochemical analysis could also be used to detect PDH deficiency (24). These methods require incubating cell cultures with antibodies that targets the α-subunit of PDH (24–26).

Nuclear magnetic resonance (NMR) spectroscopy is often used to determine PDH deficiency *via* either ^31^P or ^13^C NMR. This method investigates isotopomeric patterns of the carbons in of glutamate (27). Fibroblasts are taken from patients, cultured, and then exposed to ^13^C-glucose. The changes in glucose and lactate produced are observed. For the ^31^P NMR spectroscopy, the resonance of α-NTP and α-NDP are analysed. For the ^13^C NMR spectroscopy, the resonance of multiplet areas belonging to glutamate carbons 2, carbon 3 and carbon 4 are calculated. These areas are analysed using a line-fit analysis (27).

Radiochemical methods can also be used to determine the activity of the PDH complex by measuring the ^14^CO_2_ produced from [1-^14^C]-pyruvate (28). Using this approach, the enzymatic activity of PDH can be determined before and after treatment with dichloroacetate, an activator of the PDH complex (25). Normal fibroblasts treated with dichloroacetate should have 5–20 times the amount of ^14^CO_2_ observed in an untreated sample (29). Normal values would be around 5-6 nmol/min per mg of protein whereas a patient with PDH deficiency would show values around 0.1–0.3 nmol/min per mg of protein (29).

## Current Treatmments of Pyruvate Dehydrogenase Deficiency

The current known diet and treatments for PDH deficiency are the ketogenic diet, thiamine and dichloroacetate.

The ketogenic diet is a diet that comprises mostly fat, moderate-to-low protein and low carbohydrate (3). Clinical Global Impression of Improvement was used for assessment and this diet has shown to improve health, and is an effective option, for PDH deficiency patients (12). Although, it should also be noted that the ketogenic diet could not improve the neurological symptoms and/or reverse the brain damage (2). Consuming a ketogenic diet over a long term can yield some side effects, such as obstipation, pancreatitis, sialorrhea and vomiting (12).

Another treatment uses thiamine, a cofactor of PDH that was required for the conversion of pyruvate (30). Treatment with thiamine for PDH deficiency may only work with certain pathogenic variants on the thiamine pyrophosphate binding site within the α-subunit of PDH, i.e., p.H44R, p.R88S, p.G89S, p.R263G, and p.V389fs (31, 32).

Dichloroacetate treatment can reduce blood lactate concentrations. An ongoing phase 3 clinical trial on dichloroacetate for PDH complex deficiency commenced in 2015. Self-complementary adenovirus expressing the PDH α-subunit gene can be combined together with dichloroacetate to increase the production of ATP (33). But based on the study by Han et al., it produces a low transgene expression and therefore might be effective enough to treat the disease (33).

In addition to the treatments above, phenylbutyrate could be potential treatment for PDH deficiency. Little is known on the effect of phenylbutyrate for PDH deficiency, but similar to dichloroacetate, phenylbutyrate acts on PDH kinases (26, 34). Thus, phenylbutyrate might be effective in regulating PDH deficiency through the activity of PDH kinase. A phase 2 clinical trial for PDH complex deficiency with a single participant was conducted back in 2018 and completed in 2020. The result of this clinical trial has not been published.

Even though PDH deficiency was classified as a rare inherited disease, it is also considered a common mitochondrial disease (2). As this disease affects a child’s growth and development, it is important to have a treatment to reduce its severity at an early age. The following sections entailed the review on the use of phenylbutyrate or dichloroacetate or the combination of both in treating PDH deficiency.

### Phenylbutyrate as a Treatment for Pyruvate Dehydrogenase Deficiency Caused by *PDHA1* Pathogenic Variant

Phenylbutyrate acts on PDH kinase and not on the PDH itself. In PDH kinase 2, there are two sites for binding—near the ATP lid and a corresponding binding site of Pfz3 (35). PDH kinase has four helix bundle, these motifs make up an ATP-binding fold. Phenylbutyrate binds on the core (4-helix bundle); Val67 was found in the middle of this bundle and has a role in stabilizing the binding of phenylbutyrate (35). Phenylbutyrate binds close to the ATP lid due to its flexibility. This was shown when a nucleotide binds on the ATP lid of PDH kinase. The ATP lid would adjust itself to allow the nucleotide and its phosphate to fit into the ATP lid (10). Phenylbutyrate binding would cause a conformational change and interfere with a specific protein-protein interaction (35). This would result in interfering the binding between PDH and PDH kinase, allowing the PDH to remain active.

It was initially thought to function as a histone deacetylase inhibitor as it has been shown to induce differentiation, cell cycle arrest and apoptosis in cancer cells (36). A study was done to verify the effect of phenylbutyrate. Protein expression levels of PDH complex subunits, PDH phosphatase and PDH kinase as well as levels of the mRNA transcripts in mice brains were evaluated (26). It was concluded that, phenylbutyrate increases the activity of PDH *via* inhibition of PDH kinase activity and not by increasing the transcription level (26).

Phenylbutyrate acts as a different type of inhibitor in different isoforms of PDH kinase. With PDH kinase 2, phenylbutyrate acts as a reversible and competitive inhibitor (35). In the case of PDH kinase 1 or PDH kinase 3, phenylbutyrate acts as a non-competitive inhibitor. These properties of phenylbutyrate were observed *via* a Lineweaver Burk plot when a saturating and non-saturating concentration of phenylbutyrate was used (35). This difference lies in the structure of PDH kinase 2. In other PDH kinases, the valine found in the 4-helix bundle is replaced with leucine and phenylbutyrate was unable to bind to it. Phenylbutyrate appears to have no effect on PDH kinase 4 (35).

Phenylbutyrate could take some time for observable changes to be seen, it may not be observed in the first 2-6 h after administration, but it would be apparent approximately 24 h later (26). Not all types of pathogenic variant respond well with phenylbutyrate, most responsive pathogenic variants occur near the active site of PDH. This was because phenylbutyrate inhibits and prevents phosphorylation that normally happens within the active site (26). Other pathogenic variants located further away from the active site are not influenced by phenylbutyrate action. This could be due to their differing pathogenic variants which cause changes in oligomeric structure, the alteration of hydrophobic interactions, etc (26). To date, no studies reported the toxic side effects of phenylbutyrate in PDH deficiency patients.

In another observation reported by Ferriero et al., phenylbutyrate tended to be more responsive with proteins displaying missense pathogenic variants and unresponsive with those displaying large deletion or nonsense pathogenic variants (as shown by samples from skin fibroblast obtained from male and female patients) (10). All missense pathogenic variants of PDH in male patients found in this experiment (p.P221L, p.R234G, p.G249R, p.R349C, p.R349H) were responsive to phenylbutyrate, but p.R349R and p.R349H required 5 days of treatment in order to show an increase in enzymatic activity. The PDH p.R349A pathogenic variant also responded similarly when dichloroacetate was used. A synonymous mutation may also occur, a substitution from C to T (c.483C > T) results in p.Y132Y pathogenic variant. A patient with a PDH p.Y132Y pathogenic variant would display splicing in about 40% of the total cDNA. Incorrect splicing would usually result in a frameshift and premature termination of the protein. Phenylbutyrate will most likely increase the enzymatic activity that belongs to the remaining correctly spliced PDH enzyme. Patients with a truncating pathogenic variant (p.Q351_R378dup) showed no response to phenylbutyrate treatment (10).

There were differences in the effect of phenylbutyrate in male and female patients (10). It is important to point out that the males only have one copy of the X chromosome whereas the females have two copies—one the wild type, the other containing the pathogenic variant. It could be quite hard to determine the effect of phenylbutyrate in female patients as phenylbutyrate could increase the enzymatic activity of PDH kinase belonging to the protein populations encoded by the wild type allele. In females, most missense, mis-spliced and truncation pathogenic variants would have an impact on phenylbutyrate’s effect. Patients with the p.Q279H pathogenic variant, truncation pathogenic variants (p.E98_Q170del or p.N381SFsX43) or 2.14 Mb deletion could not be treated with phenylbutyrate. Phenylbutyrate has no effect on this pathogenic variant likely due to X-inactivation (a process where one of the X chromosomes is inactivated) in female patients. Skewed X-inactivation results in an unequal number of cells with wild type X chromosome inactivated compared to those harbouring the mutant alleles. Therefore, female patients with these particular pathogenic variants display favouritism towards the mutant over the wild type (10).

To conclude, phenylbutyrate would only be able to treat patients with small pathogenic variants close to the ATP lid of the PDH kinase. Phenylbutyrate was shown to only have effect to PDH kinase 1, PDH kinase 2 and PDH kinase 3. Phenylbutyrate could only work on certain pathogenic variants, usually caused by a missense pathogenic variant near the ATP lid.

### Dichloroacetate as a Treatment for Pyruvate Dehydrogenase Deficiency Caused by *PDHA1* Pathogenic Variant

Dichloroacetate is a structural analogue to pyruvate (37). Dichloroacetate has the same binding site as pyruvate on PDH kinase and pyruvate binds in this site as feedback for inhibition (38). Dichloroacetate binds on the PDH kinase N-terminal domain and stabilizes the PDH complex, which leads to decreased lactate and pyruvate levels in the blood and the cerebrospinal fluid (37, 39). In previous studies, its use could increase the PDH activity in both wild type skin fibroblasts and PDH deficiency patients. Unlike phenylbutyrate, dichloroacetate binds to all PDH kinase isoforms. Notably, PDH kinase 2 and PDH kinase 4 are the most sensitive to dichloroacetate inhibition, and PDH kinase 3 the least sensitive (40).

Dichloroacetate acts rapidly within minutes after its oral administration (40). The concentration of dichloroacetate administered to the body has to be considered as prolonged and repeated use of dichloroacetate could inhibit its metabolism and it could also cross the blood brain barrier (41–43). Two of the studies highlight the importance of dosing with dichloroacetate (37, 44). The metabolism of dichloroacetate could have a toxic effect on testicular tissue and also may cause hepatomegaly and induce birth defects (37). Another example would be from a 1-year old patient who was administered with dichloroacetate combined with vitamin B complex and lipoic acid. Within 10 days there was an indication that she was developing cholestasis (44). In adults, dichloroacetate was associated with hepatocellular and peripheral nerve toxicity (26). Neurotoxicity may arise due to the reduction and dehalogenation of dichloroacetate, forming monochloroacetate, a compound that may cause neurotoxicity (42).

To conclude, dichloroacetate was shown to have an effect on PDH kinase 2, PDH kinase 4 and PDH kinase 3. Administering dichloroacetate could reduce the lactic acidosis but not resolve the neurological problems and other clinical outcomes (42). Furthermore, dichloroacetate could be combined with a self-complementary adeno-associated virus vector that delivers and expresses the α-subunit of PDH (33). The delivery of the vector alone causes a slight increase in PDH activity. It is thought that dichloroacetate increases the stability of the complex, which will eventually allow more PDH enzyme to be dephosphorylated (33).

### Phenylbutyrate and Dichloroacetate as a Treatment for Pyruvate Dehydrogenase Deficiency Caused by *PDHA1* Pathogenic Variant

Phenylbutyrate and dichloroacetate bind on different sites of PDH kinase and so the combination of these two treatments is theoretically possible. A study by Ferriero et al., uses a mouse model to investigate the combination of these two treatments, a dose of 250 mg/kg/day of phenylbutyrate and dichloroacetate each was administered (35). The concentration of dichloroacetate used in this study was higher compared to the amount administered to cancer patients (25 mg/kg/day) but it produced a better result compared to phenylbutyrate or dichloroacetate alone (35).

Based on the PDH complex activity, the combination of these two treatments therefore showed promising results in mice, but there was no comparable data available for a similar combination therapy in humans. It could potentially produce a different effect in humans as the amount of dichloroacetate used in the experiment was 10x higher. Reports showed that a saturating amount of dichloroacetate might inhibit its metabolism and cause side effects (37, 42, 44). Combination therapy has potential in handling or ameliorating PDH deficiency as it seemed to have a positive result as seen on the murine model by Ferriero et al. However, more studies need to be done on the effect of dichloroacetate and phenylbutyrate on the different PDH kinase isoforms as conformational changes may yield different result.

## Which Treatment Works Best for Pyruvate Dehydrogenase Deficiency Caused by the *PDHA1* Gene?

**FIGURE 2:**
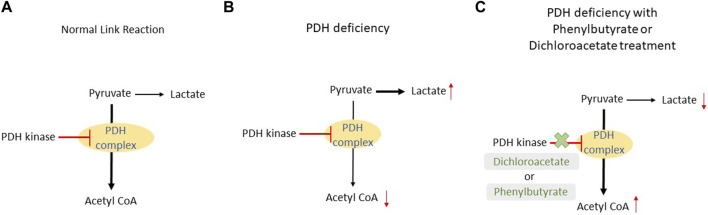
Comparison between a normal link reaction, link reaction in patients with PDH deficiency and in the presence of dichloroacetate or phenylbutyrate. **(A)** In a normal patient, pyruvate is mostly converted to acetyl CoA with the aid of the PDH complex. This reaction is inhibited by PDH kinase once ATP is produced in the subsequent reaction. **(B)** In PDH deficiency patients, a pathogenic variant in the PDH complex causes a slower conversion of pyruvate to acetyl CoA. As a result, pyruvate will build up and it will be converted to lactate. **(C)** The addition of dichloroacetate or phenylbutyrate will inhibit the PDH kinase and this will prolong the activity of the PDH complex; more acetyl CoA will be produced.

Dichloroacetate and phenylbutyrate allow for a prolonged activation of the PDH complex, so more pyruvate is turned to acetyl CoA (Figure 2). When these treatments are compared, it appears that dichloroacetate has an advantage. Theoretically, dichloroacetate will have an effect on most enzymes displaying pathogenic variants for PDH deficiency as it directly interacts with the same site as pyruvate. In comparison, phenylbutyrate would only be effective in patients with a certain type of pathogenic variant that do not impact its binding area. Phenylbutyrate showed results that it could reduce the severity of PDH deficiency but, again, depends on what type of pathogenic variant the patient has. Table 1 summarizes the studies that used dichloroacetate and/or phenylbutyrate as treatment for PDH deficiency.

**TABLE 1:** Summary of dichloroacetate and/or phenylbutyrate as treatment for PDH deficiency due to *PDHA1* pathogenic variant.

Type of Treatment	Reference	Type of study	Summary
Phenylbutyrate	(26)	Murine and zebrafish model	Phenylbutyrate increased the PDH complex activity in the brain. The brain is particularly important in the murine model for PDH deficiency as it showed a lot of morphological and histological changes. Different genders have different types of pathogenic variants
		Human fibroblast with PDH deficiency	
	(10)	Human fibroblast with PDH deficiency	The p.P221L, p.R234G, p.G249R and p.Y132Y pathogenic variants in male patients responded to phenylbutyrate. The p.R349C and p.R349H did not produce a good response towards phenylbutyrate. The p.R349C pathogenic variant, in particular, took 5 days to produce a response
	(35)	Murine model	Phenylbutyrate bound to 2 sites of PDH kinase 2: the ATP lid and the binding site of Pfz3. Phenylbutyrate was noncompetitive inhibitors of PDH kinase 1 and PDH kinase 3. It did not affect PDH kinase 4. PDH kinase 2 and PDH kinase 3 bound stronger than PDH kinase1
		Human fibroblast with PDH deficiency	
Dichloroacetate	(37)	Review	Dichloroacetate had toxic effect(s) on testicular tissue and also might cause hepatomegaly and induce birth defects
	(44)	Observation study of a female patient with PDH deficiency	Administration of dichloroacetate along with vitamin B complex and lipoic acid caused the development of cholestasis
	(26)	Murine and zebrafish model	Dichloroacetate stabilized the enzyme. It reduced the blood, cerebrospinal fluid and brain lactate concentration. Peripheral neuropathy and hepatocellular toxicity might occur
		Human fibroblast with PDH deficiency	
	(33)	Human fibroblast with PDH deficiency	Combination of dichloroacetate and self-complementary adeno-associated virus vector that delivered and expressed the α-subunit of PDH, caused a slight increase in PDH activity
	(42)	Observation study of 43 patients with congenital lactic acidosis. 11 of them were diagnosed with PDH deficiency	Generally, dichloroacetate reduced the severity of congenital lactic acidosis, but there was no improvement in neurologic problem and other clinical outcomes
	(40)	Review	PDH kinase 2 and 4 were the most sensitive kinases to dichloroacetate inhibition, followed by PDH kinase 1 and lastly, PDH kinase 3
	(39)	Observation study of an patient with PDH α-subunit pathogenic variant	Dichloroacetate bound to the N-terminal domain of PDH kinase and stabilized the PDH complex
Phenylbutyrate and Dichloroacetate	(35)	Human fibroblast with PDH deficiency	Combination treatment resulted in a greater reduction in phosphorylation of the α-subunit of PDH compared to the individual treatment

For clarity, dichloroacetate is only used to reduce the severity of lactic acidosis as it has shown no improvement of the neurological issues and other clinical outcomes (42). Once a proper diagnosis of the disease is made, dichloroacetate could be exchanged or combined with other more appropriate treatments. Treatment choice would depend on where the pathogenic variant is located within PDH. For pathogenic variants near the ATP binding site such as p.P221L, p.R234G, p.G249R, p.R349C, p.R349H, phenylbutyrate would be a better option. Alternatively, patients with p.H44R, p.R88S, p.G89S, p.R263G, and p.V389fs pathogenic variants in the α-subunit of PDH could use thiamine as a treatment (32). There is clearly a need for more treatment options for other pathogenic variant sites as phenylbutyrate is not compatible with other types of pathogenic variant (phenylbutyrate does not have an effect on certain truncation pathogenic variants (i.e., p.Q351_R378dup) and pathogenic variants such p.R349R and p.R349H lead to a delay in the treatment producing a result). Other PDH kinase inhibitors could be a potential treatment option as dichloroacetate and phenylbutyrate are both PDH kinase inhibitors and drugs that enhances PDH phosphatase activity to prolong the activation of PDH may also be a potential treatment for future consideration.

## Limitations

The side effects of phenylbutyrate and dichloroacetate treatment is not fully understood. A study by Kaufmann et al. (45) explained the possible side effects of dichloroacetate in adults such as peripheral nerve toxicity but did not explain its side effect in other age groups. As this inborn error of metabolism is an early onset disease, it is natural that the treatment of children is a primary concern. Potential side effects in children need to be understood as there is a chance that these treatments act differently in different age groups. Considering PDH deficiency impacts brain development, side effects could exacerbate this in a way that may be irreversible and affect the person for a lifetime. In order to address these questions, further animal models need to be developed and be used to increase understanding of potential benefits versus side effects before clinical trials.

Dosing is another matter that needs to be investigated. Hypothetically, if a male and a female patient has the same site of pathogenic variant and is responsive to phenylbutyrate, the female patient would most likely show a better result as about half of the PDH enzyme is still functioning normally. If that is the case, could there be a dosing adjustment for female patients with this disease? It is hoped that dosing adjustment would reduce the side effects produced by the treatment.

Once a proper diagnosis is given and the pathogenic variant is known, a more suitable treatment could be selected. However, the biochemical diagnosis method for PDH deficiency can be unreliable as it depends on the consumption of carbohydrates to check for the activity of PDH. The time taken to detect this disease is also time consuming, for example, the skin fibroblast test that measures the ^14^CO_2_ formed as well as the immunoblot test. A more reliable method of diagnosis for example targeted exome sequencing should be proposed to decrease the possibility of childhood mortality.

## Conclusion

Phenylbutyrate is similar to thiamine in the case of PDH deficiency. Only patients with pathogenic variants close to the ATP lid such as p.P221L, p.R234G, p.G249R, p.R349C, p.R349H, would be responsive to phenylbutyrate. Dichloroacetate on the other hand could be used to reduce lactic acidosis but in high concentration, it runs the risk of producing harmful side effects. Theoretically dichloroacetate will have an effect on all sorts of pathogenic variants as it is an analogue of pyruvate. More studies involving cell culture, animal models and clinical trials are needed to fully understand PDH deficiency and its treatment.

## Abbreviation

PDH, pyruvate dehydrogenase.
